# Supplementary material for: What is good grief support? Exploring the actors and actions in social support after traumatic grief
Source: PLoS One. 2021 May 27;16(5):e0252324. doi: 10.1371/journal.pone.0252324 (PMC8158955; doi:10.1371/journal.pone.0252324)
Supplement: S1 Questionnaire — (DOCX) [file pone.0252324.s001.docx]

Defining and understanding social support in grief

Survey Flow

Block: Default Question Block (51 Questions)

| Page Break |  |
| --- | --- |

Start of Block: Default Question Block

Q1
Principal Investigator:  Dr. Joanne CacciatoreCo-investigator: Lori Barnes, ND Information:  You are invited to participate in a research study. Before you enroll to be a research participant, it is important that you read the following information and ask as many questions as necessary to be sure that you understand what your participation will involve. You must be over the age of 18.

Purpose:This research study explores what is helpful social support to people who are grieving the death of a spouse, child, parent, or sibling. Procedures: - Participants will answer an online survey.

- The questionnaire will take about 30-45 minutes. Questions will relate to social support from  friends, family and professionals and also substance use. Any question can be skipped.

- Participants may also be randomly selected for a 30 minute phone interview. For those who  consent to the phone interview we are asking your permission to audio record the interview.  Only the research team will have access to the recordings. The recordings will be deleted  immediately after being transcribed and any published quotes will be anonymous. To protect  your identity, please refrain from using names or other identifying information during the  interview. Let us know if, at any time, you do not want to be recorded and we will stop the   recording and take hand notes instead.

- Results will be made available to research participants upon completion should you desire to   provide your email.

Inclusion Requirements: Those above the age of 18 who have lost a spouse, child, sibling or parent.

Voluntary Participation: *Participation in this study is voluntary. You may refuse to participate or withdraw from the study at any time. You can also skip any questions you do not wish to answer.*You will be informed of any significant new information regarding this study that may affect your willingness to continue.

Confidentiality: *The records of your participation are confidential and the results will be double password protected in an online database. This study may result in scientific presentations and publications, but steps will be taken to ensure you are not identified by name.*

Questions: *For more information concerning this study and research-related risks or injuries, you may contact the Principal Investigator, Dr. Joanne Cacciatore at joanne.cacciatore@asu.edu or 602-574-1000. If you have any questions about your rights as a subject/participant in this research, or if you feel you have been placed at risk, you can contact the Chair of the Human Subjects Institutional Review Board, through the  ASU Office of Research Integrity and Assurance, at (480) 965-6788.
It is important for the veracity of the research study to answer all*questions please.*By proceeding to the next question, you have given consent to*participate*and we thank you.*

Are you

- Male (1)
- Female (2)
- Non binary (3)

What is your ethnic heritage?

- White (1)
- Black or African American (2)
- American Indian or Alaska Native (3)
- Asian (4)
- Native Hawaiian or Pacific Islander (5)
- Other (6)

What is your highest education level?

- Less than high school (1)
- High school graduate (2)
- Some college (3)
- 2 year degree (4)
- 4 year degree (5)
- Professional degree (6)
- Doctorate (7)

What is your general household annual income?

- Less than $10,000 (1)
- $10,000 - $19,999 (2)
- $20,000 - $29,999 (3)
- $30,000 - $39,999 (4)
- $40,000 - $49,999 (5)
- $50,000 - $59,999 (6)
- $60,000 - $69,999 (7)
- $70,000 - $79,999 (8)
- $80,000 - $89,999 (9)
- $90,000 - $99,999 (10)
- $100,000 - $149,999 (11)
- More than $150,000 (12)

What is the name of your loved one who died?

________________________________________________________________

What is your relationship to the person who died?

- My child (1)
- My spouse/partner (2)
- My parent (3)
- My sibling (4)
- None of the above (5)

Skip To: End of Survey If What is your relationship to the person who died? = None of the above

When did he or she die?

- Within the last year (1)
- 1-3 years ago (2)
- 3-5 years ago (3)
- More than 5 years ago (4)

What was your loved one's cause of death?

- Illness or disease (1)
- Accident (2)
- Suicide (3)
- Homicide (4)
- Perinatal death related causes (5)
- Unknown/undetermined (6)
- Other (7) ________________________________________________

Age of your loved one at the time of his or her death?

- Stillborn to 3 (1)
- 3-11 (2)
- 12-17 (3)
- 18-24 (4)
- 25-40 (5)
- 41-55 (6)
- 56-70 (7)
- Over 70 (8)

Are you currently taking any medications?

- Yes (1)
- No (2)

Skip To: Q15 If Are you currently taking any medications? = No

What kinds of medications are you currently taking?

- Psychiatric (i.e. antidepressants, sleep aids, anti-anxiety meds, antipsychotics) (1)
- Heart medications (2)
- Pain medications (3)
- Thyroid (4)
- Cholesterol lowering medications (5)

Skip To: If What kinds of medications are you currently taking? = Psychiatric (i.e. antidepressants, sleep aids, anti-anxiety meds, antipsychotics)

This next question is two part. Please list the names of your medications and how long you've been taking them:

________________________________________________________________

What is your partner status?

- Married (1)
- Widowed (2)
- Divorced (3)
- Separated (4)
- Single (5)
- Partnered (6)

How do you feel your social support has been since your loved one died?

- Very poor (1)
- Poor (2)
- Adequate (3)
- Good (4)
- Excellent (5)

What does social support in grief mean to you?

________________________________________________________________

What kinds of actions or responses felt supportive to you?

________________________________________________________________

What kinds of actions or responses felt unsupportive to you?

________________________________________________________________

How might others in your social support system do better to help you?

________________________________________________________________

If applicable in each category, how satisfied are you with the crisis care you received during the initial period after your loss from these groups?

|  |  |
| --- | --- |
| Nursing staff (1) | ▼ Extremely satisfied (1) ... N/A (6) |
| Physicians (2) | ▼ Extremely satisfied (1) ... N/A (6) |
| Hospital social worker (3) | ▼ Extremely satisfied (1) ... N/A (6) |
| Funeral staff (4) | ▼ Extremely satisfied (1) ... N/A (6) |
| Faith leaders (5) | ▼ Extremely satisfied (1) ... N/A (6) |
| First responders/paramedics (6) | ▼ Extremely satisfied (1) ... N/A (6) |
| Crisis response teams (7) | ▼ Extremely satisfied (1) ... N/A (6) |
| Hospice workers (8) | ▼ Extremely satisfied (1) ... N/A (6) |
| Law enforcement (9) | ▼ Extremely satisfied (1) ... N/A (6) |

If applicable in each category, how satisfied are you with the overall social support since your loss that you have received from any of these groups?

|  |  |
| --- | --- |
| Counselors or therapists (1) | ▼ Extremely satisfied (1) ... N/A (7) |
| Faith leaders (2) | ▼ Extremely satisfied (1) ... N/A (7) |
| Family (3) | ▼ Extremely satisfied (1) ... N/A (7) |
| Friends (4) | ▼ Extremely satisfied (1) ... N/A (7) |
| Neighbors/community (5) | ▼ Extremely satisfied (1) ... N/A (7) |
| Online grief groups (6) | ▼ Extremely satisfied (1) ... N/A (7) |
| In person grief groups (7) | ▼ Extremely satisfied (1) ... N/A (7) |
| Colleagues (11) | ▼ Extremely satisfied (1) ... N/A (7) |
| Pets/animals (12) | ▼ Extremely satisfied (1) ... N/A (7) |

The next set of questions specifically explores your social support experiences.


If I wanted to visit my loved one at the cemetery, I would have a hard time finding someone to go with me.

- Definitely false (1)
- Probably false (2)
- Probably true (3)
- Definitely true (4)

I feel there is no one with whom I can share my most private worries and fears.

- Definitely false (1)
- Probably false (2)
- Probably true (3)
- Definitely true (4)

When I'm intensely grieving, I can easily find someone to help me with my daily chores.

- Definitely false (1)
- Probably false (2)
- Probably true (3)
- Definitely true (4)

There's someone I can turn to for advice if problems arise in my family.

- Definitely false (1)
- Probably false (2)
- Probably true (3)
- Definitely true (4)

If I decided one afternoon that I needed company, I could easily find someone to spend time with me.

- Definitely false (1)
- Probably false (2)
- Probably true (3)
- Definitely true (4)

When I need support dealing with grief, I know someone I can turn to.

- Definitely false (1)
- Probably false (2)
- Probably true (3)
- Definitely true (4)

I don't often get invited to do things with others.

- Definitely false (1)
- Probably false (2)
- Probably true (3)
- Definitely true (4)

If I had to go out of town for a few weeks, it would be difficult to find someone to look after my house/apartment (plants, pets, garden, etc).

- Definitely false (1)
- Probably false (2)
- Probably true (3)
- Definitely true (4)

If I wanted to have lunch with someone, I could easily find someone to join me.

- Definitely false (1)
- Probably false (2)
- Probably true (3)
- Definitely true (4)

If I was stranded 10 miles from home, there is someone I could call to come and get me.

- Definitely false (1)
- Probably false (2)
- Probably true (3)
- Definitely true (4)

It is difficult to find someone who can support me in my grief.

- Definitely false (1)
- Probably false (2)
- Probably true (3)
- Definitely true (4)

If I needed help dealing with my loved one's belongings (his or her room, personal items), I would have a hard time finding someone to help me.

- Definitely false (1)
- Probably false (2)
- Probably true (3)
- Definitely true (4)

Please answer the following question/s honestly, remembering this survey is completely anonymous.  


Do you ever consume alcohol or recreational drugs?

- Yes (1)
- No (2)

Skip To: If Please answer the following question/s honestly, remembering this survey is completely anonymous.... = No

Do you find yourself thinking about when you will be able to have a drink or take drugs?

- Never (1)
- Sometimes (2)
- Often (3)
- Nearly always (4)

Is drinking or taking drugs more important than anything else you might do during the day?

- Never (1)
- Sometimes (2)
- Often (3)
- Nearly always (4)

Do you feel your need to drink or use drugs is too strong to control?

- Never (1)
- Sometimes (2)
- Often (3)
- Nearly always (4)

Do you plan your days around getting/taking alcohol or drugs?

- Never (1)
- Sometimes (2)
- Often (3)
- Nearly always (4)

Do you drink or take drugs in a particular way to increase the effects?

- Never (1)
- Sometimes (2)
- Often (3)
- Nearly always (4)

Do you drink or use drugs morning, afternoon, and evening?

- Never (1)
- Sometimes (2)
- Often (3)
- Nearly always (4)

Do you feel you have to continue using drugs or alcohol once you've started?

- Never (1)
- Sometimes (2)
- Often (3)
- Nearly always (4)

Is getting an effect more important than the particular drink or drug you use?

- Never (1)
- Sometimes (2)
- Often (3)
- Nearly always (4)

Do you want to use more once the effects start to wear off?

- Never (1)
- Sometimes (2)
- Often (3)
- Nearly always (4)

Do you find it difficult to cope without drugs or alcohol?

- Never (1)
- Sometimes (2)
- Often (3)
- Nearly always (4)

Is there anything else you'd like us to know?

________________________________________________________________

Are you willing to participate in a brief, 30 mins phone interview about your experiences if randomly selected?

- Yes (1)
- No (2)

Skip To: Q27 If Are you willing to participate in a brief, 30 mins phone interview about your experiences if rand... = Yes

Please provide an email where we can contact you:

________________________________________________________________

Thank you so very much for participating in this important study. We hope to improve social support for grieving people, and you've helped us begin to understand how. 


If you need extra support, please consider reaching out:

The Compassionate Friends
www.TCF.org
TCF provides support groups for bereaved parents. 

Warfighter Advance
www.WarfighterAdvance.org
WFA provides supportive services and retreats for veterans who are traumatized by loss, combat, and are facing emotional distress.

Parents of Murdered Children
www.POMC.org
POMC provides legal and emotional support to parents and families when a family member is murdered.

Soaring Spirits
www.soaringspirits.org
Soaring Spirits provides support for widowed people.

End of Block: Default Question Block
