# Supplementary material for: What is good grief support? Exploring the actors and actions in social support after traumatic grief
Source: PLoS One. 2021 May 27;16(5):e0252324. doi: 10.1371/journal.pone.0252324 (PMC8158955; doi:10.1371/journal.pone.0252324)
Supplement: Attachment — Submitted filename: Response to Reviewers- PONE-D-21-04230R1.docx [file pone.0252324.s003.docx]

March 18, 2021

Dear Dr. Fernández-Alcántara:

Thank you and the reviewers for the helpful feedback to improve this important manuscript. We are pleased to submit what we feel is a much-improved version of the manuscript.

In answer to each review, please see inline below:

Reviewer #1:  We appreciate your helpful comments and drawing our attention to some areas needing attention. We revisited the methodology section, ensuring to emphasize the objectives, design, and methods. Participant demographics are detailed in results section. We are happy to move participant demographic data to the methods section however this seems an unusual placement for these data to us. The research study took place in January of 2020 (detailed in the first version of the manuscript) however we clarified that the loneliness of grief, exacerbated by the pandemic, is one source of justification for the importance of this study.

Reviewer #2: We are glad you found this to be an interesting paper. To address R2’s concerns, we added more detail on QA and content analysis in the abstract. We added titles to the tables. We added investigator triangulation (Denzin, 1978) to clarify the type. The subject of gender in grief is certainly interesting; however, there is not enough data to sufficiently address this issue in a meaningful way. We added comments about this in the limitations. We also changed the “implications” section to “conclusion”.

Reviewer #3: Thank you for your helpful feedback. We removed the comment that this study is part of a larger study. It is, indeed, but these data are the larger data; from this other studies will emerge. We can see how this line was confusing. Thank you. We clarified the population (inclusion) in the methods section. We agree wholeheartedly that more specific research on group-needs is warranted, such as lifespan and sex/gender. We are hoping this study will inspire more precise future research. We also added a comment in the limitations section on lifespan. Thank you. We clarified the survey question related to both acute and long-term grief support in the method section. Participants themselves determined acute vs long term support; however, the list of likely providers also fit into the period post loss. For example, first responders do not provide long term care and are in the ‘acute’ care category. Some fit both categories (i.e. family). With regards to social workers, physicians, law enforcement, and faith leaders: prior research indeed indicates that provider burn out may be an issue. However, we were unable to find research to support the vicarious trauma/burnout rationale for better outcomes of support between funeral directors and hospice workers (who scored high and experience intense demands that are emotionally laborious) and (for example) faith leaders and hospital social workers (who scored low and who also experience high demands and are emotionally laborious).

We appreciate the feedback very much. Thank you for giving us a chance to improve this manuscript.

Sincerely,

Joanne Cacciatore, PhD; Kara Thieleman, PhD; Ruth Fretts, MD; Lori Barnes.
